# Iowa State Medical Society

**Published:** 1856-06

**Authors:** 


### IOWA STATE MEDICAL SOCIETY.

This society held its annual meeting at Ottumwa recently' the proceedings of which, we are unable to publish in this issue. We were glad to observe, when in attendance, there, that the interest was marked in behalf of the well being of the society. A large addition to the membership of some of the best medical men of the State, was a guaranty of this. It was re-organized with a new and a better constitution and no doubt was felt, but that the society, which is now over six years in existence, will rank high among the State organizations of the country. Strong resolutions in favor of the "Old University School" was passed without a dissenting vote and the warmest expressions of zeal were indulged toward the "Iowa Medical Journal" by gentlemen of high standing in the profession. A large addition to the subscription was obtained. The next meeting will take place at Iowa City, when we shall expect to see the largest medical meeting ever convened in the State. We hope we will not be disappointed in this hope.

The proceedings next number.

